# Herg K^+^ Channel-Dependent Apoptosis and Cell Cycle Arrest in Human Glioblastoma Cells

**DOI:** 10.1371/journal.pone.0088164

**Published:** 2014-02-06

**Authors:** Ingo Staudacher, Julian Jehle, Kathrin Staudacher, Hans-Werner Pledl, Dieter Lemke, Patrick A. Schweizer, Rüdiger Becker, Hugo A. Katus, Dierk Thomas

**Affiliations:** 1 Department of Cardiology, University Hospital Heidelberg, Heidelberg, Germany; 2 Department of Neonatology, University Hospital Heidelberg, Heidelberg, Germany; 3 Department of Neurooncology, University Hospital Heidelberg, Heidelberg, Germany; 4 German Cancer Consortium Clinical Cooperation Unit Neurooncology, German Cancer Research Center, Heidelberg, Germany; University of Miami School of Medicine, United States of America

## Abstract

Glioblastoma (GB) is associated with poor patient survival owing to uncontrolled tumor proliferation and resistance to apoptosis. Human ether-a-go-go-related gene K^+^ channels (hERG; Kv11.1, *KCNH*2) are expressed in multiple cancer cells including GB and control cell proliferation and death. We hypothesized that pharmacological targeting of hERG protein would inhibit tumor growth by inducing apoptosis of GB cells. The small molecule hERG ligand doxazosin induced concentration-dependent apoptosis of human LNT-229 (EC_50_ = 35 µM) and U87MG (EC_50_ = 29 µM) GB cells, accompanied by cell cycle arrest in the G0/G1 phase. Apoptosis was associated with 64% reduction of hERG protein. HERG suppression *via* siRNA-mediated knock down mimicked pro-apoptotic effects of doxazosin. Antagonism of doxazosin binding by the non-apoptotic hERG ligand terazosin resulted in rescue of protein expression and in increased survival of GB cells. At the molecular level doxazosin-dependent apoptosis was characterized by activation of pro-apoptotic factors (phospho-erythropoietin-producing human hepatocellular carcinoma receptor tyrosine kinase A2, phospho-p38 mitogen-activated protein kinase, growth arrest and DNA damage inducible gene 153, cleaved caspases 9, 7, and 3), and by inactivation of anti-apoptotic poly-ADP-ribose-polymerase, respectively. In summary, this work identifies doxazosin as small molecule compound that promotes apoptosis and exerts anti-proliferative effects in human GB cells. Suppression of hERG protein is a crucial molecular event in GB cell apoptosis. Doxazosin and future derivatives are proposed as novel options for more effective GB treatment.

## Introduction

Glioblastoma (GB) is the most common malignant primary brain tumor in adults. Current treatment is based on maximal safe surgical resection, followed by chemo- and radiotherapy when feasible [1]. However, outcome is poor despite optimal therapy with a mean survival rate of 1 year following diagnosis, which is due to uncontrolled tumor proliferation, infiltrative growth, angiogenesis, and resistance to apoptosis and medical treatment [2], [3]. Thus, effective therapy of GB still remains an unmet medical need.

The human ether-a-go-go-related gene potassium channel (hERG; Kv11.1, *KCNH*2) contributes to cardiac action potential repolarization. Electrical dysfunction of the voltage-sensitive ion channel is associated with ventricular arrhythmia [4]–[11]. Moreover, hERG channels control cell proliferation and apoptosis [12]. Targeting of hERG channels by the small molecule α_1_-adrenoceptor antagonist doxazosin induces apoptosis *in vitro* independent of its anti-adrenergic function [13]–[15]. This pro-apoptotic mechanism of action was extended to structurally unrelated compounds, suggesting broader significance [11], [16]. In addition to the heart, hERG K^+^ channels are expressed in multiple types of cancer cells including GB (reviewed in [12]).

Given that GB cells express hERG channels and that hERG suppression by doxazosin induces apoptosis, we hypothesized that pharmacological targeting of hERG channels would induce apoptosis of GB cells. To explore hERG-associated GB cell apoptosis and underlying molecular pathways, human glioblastoma cell lines (LNT-229 and U87MG [17], [18]) and the hERG inhibitor doxazosin were employed. Doxazosin triggered apoptosis and caused cell cycle arrest of GB cells. Suppression of hERG protein expression *via* siRNA-mediated knock down mimicked pro-apoptotic effects of doxazosin. HERG receptor binding competition of doxazosin and the small molecule compound terazosin that had no apparent effect on cell viability attenuated doxazosin-induced apoptosis of GB cells. In summary, a hERG-dependent pro-apoptotic pathway is revealed in human glioblastoma cells, providing a novel therapeutic opportunity for future treatment of GB.

## Materials and Methods

### Cell Culture

Human LNT-229 [18] and U87MG [17] glioblastoma cells were cultered in Dulbecco’s Modified Eagle Medium (DMEM, Gibco BRL, Rockville, IL, USA) supplemented with 10% fetal calf serum (FCS), 100 U/ml penicillin G sodium, 100 µg/ml streptomycin sulphate in an atmosphere of 95% humidified air and 5% CO_2_ at 37°C. Cells were passaged regularly and subcultured prior to treatment. Drugs were added prior to analyses as indicated.

### TUNEL Staining

Apoptosis was detected by terminal deoxyribonucleotide transferase-mediated dUTP nick end labeling (TUNEL) as described [19]–[21]. Following exposure to doxazosin for 24 h, cells grown in 12-well tissue culture dishes were fixed and TUNEL reaction mixture (Roche Applied Science, Mannheim, Germany) was added to the sections according to the manufacturer’s instructions, followed by incubation at 37°C for 60 min. After removal of the TUNEL reagent slides were rinsed with phosphate-buffered saline (PBS), and TUNEL-positive cells were evaluated using a fluorescence microscope (IX 50; Olympus, Hamburg, Germany).

### XTT Cell Viability Assay

Cell viability was quantified using an assay that utilizes the ability of live cells to reduce 2,3-bis-(2-methoxy-4-nitro-5-sulfophenyl)-2H-tetrazolium-5-carboxanilide salt (XTT) to produce a colored formazan compound. Cells grown in 96-well tissue culture dishes were transferred into drug-free media after 24 h drug application. XTT (125 mM; AppliChem, Darmstadt, Germany) was then added as reported [19], and cells were maintained at 37°C and 5% CO_2_ for 2 hours in accordance with the manufacturer’s instructions. Adsorption was determined at 450 nm using a spectrophotometer (PHOmo, Anthos Mikrosysteme, Krefeld, Germany) and normalized to control measurements obtained from corresponding cells cultured in drug-free medium.

### Annexin V-FITC Apoptosis Assay

The annexin V-fluorescein isothiocyanate (FITC) assay was employed to quantify apoptosis at an early stage. Annexin V binds to phophatidylserine (PS) that is translocated to the outer leaflet of the plasma membrane during apoptosis. In addition, propidium iodide (PI) staining was applied as marker indicating compromised plasma membranes of late apoptotic LNT-229 cells. Following experimental treatment cells were rinsed with PBS, harvested using accutase (PromoCell, Heidelberg, Germany), and stained for 10 min at room temperature in the dark according to the manufacturer’s instructions (Annexin V-FITC Detection Kit; PromoCell). Cell fluorescence was detected by flow cytometry (FACScan, Becton Dickinson, Franklin Lakes, NJ, USA) and analyzed using CellQuest software (Becton Dickinson).

### Fluorocytometric Cell Cycle Analysis

Different phases of the cell cycle were distinguished by flow cytometry [22]. The assay is based on stoichiometric binding of propidium iodide to increasing amounts of DNA in cell cycle phases G0/G1, S, and G2/M. After doxazosin treatment, LNT-229 cells were trypsinized, fixed and permeabilized using ethanol, rinsed in phosphate-buffered saline (PBS), and treated with RNase A to remove RNAs. DNA was then quantitatively stained with propidium iodide for 1 h at room temperature protected from light. Fluorescence was analyzed using a FACSCanto flow cytometer (Becton Dickinson) and FlowJo software (Treestar, Ashland, OR, USA). DNA histogram data were fit with the Dean-Jett-Fox model.

### Western Blot Analysis

Protein immunodetection was performed by sodium dodecyl sulfate (SDS) gel electrophoresis and Western blotting [21], [23], [24]. LNT-229 cells were solubilized for 20 minutes at 4°C in radioimmunoprecipitation assay (RIPA) lysis buffer containing SDS and sodium deoxycholate supplemented with “Complete” protease inhibitors and “PhosSTOP” phosphatase inhibitors (Roche Diagnostics, Mannheim, Germany). Nitrocellulose membranes were developed by sequential exposure to blocking reagent (5% dry milk), primary antibodies directed against hERG (1∶200; APC-016, Alomone Labs, Jerusalem, Israel), EphA2 (1∶100; sc-924, Santa Cruz Biotechnology, Heidelberg, Germany), phospho-EphA2/Tyr-593 (1∶1,000; CB4368, Cell Applications, San Diego, CA, USA), growth arrest and DNA damage inducible gene 153 (GADD153; 1∶500; ab11419, Abcam), p38 mitogen-activated protein kinase (MAPK; 1∶1,000; 9212, Cell Signaling), phospho-p38 MAPK/Thr-180/Tyr-182 (1∶1,000; 9211, Cell Signaling), caspase 3 (1∶1,000; 9662, Cell Signaling), cleaved caspase 3 (1∶1,000; 9664, Cell Signaling), caspase 7 (1∶1,000; 9492, Cell Signaling), cleaved caspase 7 (1∶1,000; 9491, Cell Signaling), caspase 9 (1∶1,000; ab32539, Abcam), cleaved caspase 9 (1∶1,000; ab2324, Abcam), microtubule-associated protein 1 light chain 3 (LC3)A/B (1∶1,000; 4108, Cell Signaling), cleaved poly-ADP-ribose-polymerase (PARP; 1∶1000; 5625, Cell Signaling), or glyceraldehyde-3-phosphate dehydrogenase (GAPDH; 1∶40,000; G8140-11, US Biological, Swampscott, MA, USA), and appropriate horseradish peroxidase-conjugated secondary antibodies (Abcam). Signals were developed using the enhanced chemiluminescence assay (GE Healthcare, ECL Western Blotting Reagents, Buckinghamshire, UK) and quantified with ImageJ 1.46 Software (National Institute of Health, Bethesda, MD, USA). Protein content was normalized to GAPDH for quantification of optical density.

### Small Interfering RNA Knock Down

Anti-human ether-a-go-go-related gene (hERG; Kv11.1) siRNA (sc-42498; Santa Cruz) was used to knock down hERG protein expression. Scrambled siRNA (sc-37007; Santa Cruz) served as control. LNT-229 cells were allowed to settle in 6-well tissue culture dishes and antibiotic-free DMEM for 24 hours. SiRNAs (10 µmol/L stock solutions) were then transfected according to the manufacturer’s recommendation. After 9 hours, DMEM supplemented with 20% fetal bovine serum (FBS) and 2% penicillin/streptomycin was added. Following incubation for 24 hours doxazosin was added and cell viability was quantified 24 h later using the XTT assay.

### Drugs

Doxazosin, desipramine and terazosin (Sigma-Aldrich, St. Louis, MO, USA) were prepared as 10 mM stock solutions in dimethyl sulfoxide (DMSO) and stored at −20°C.

### Statistics

Data are presented as mean ± standard error of the mean (SEM) of n experiments. Statistical differences of continuous variables were determined with Origin 6 software (OriginLab, Northampton, MA, USA) using unpaired Student's *t* tests (two-sided tests). Statistical analyses were carried out prior to normalization of data. *P*<0.05 was considered statistically significant.

## Results

### The Small Molecule Compound Doxazosin Induces Apoptosis of Human LNT-229 Glioblastoma Cells

**Figure 1:**
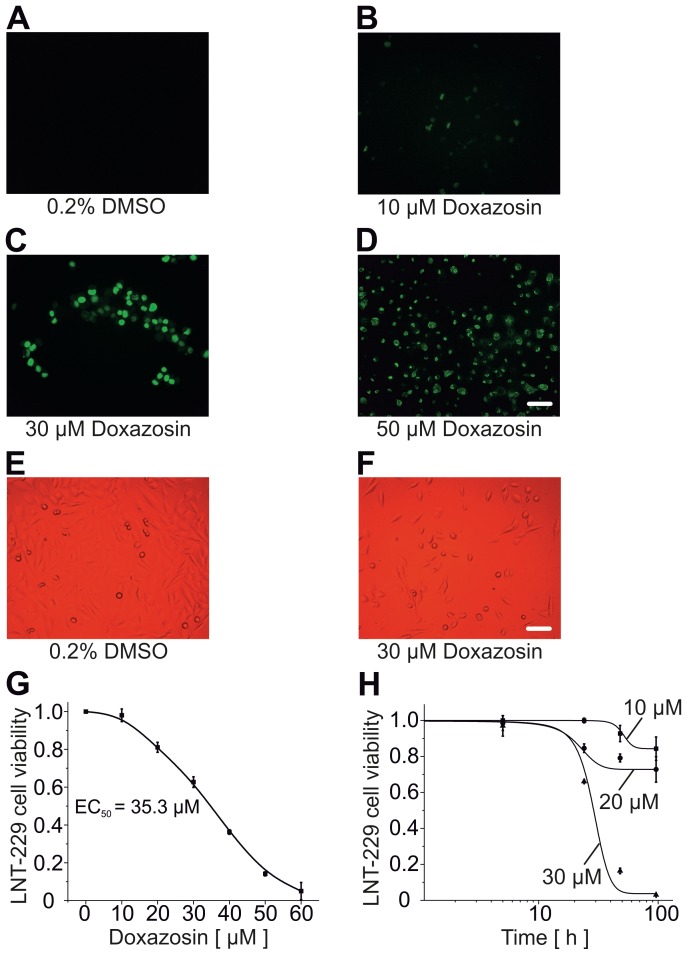
Pro-apoptotic effects of doxazosin in human glioblastoma cells. (A–D) Fluorescence microphotographs corresponding to terminal deoxyribonucleotide transferase-mediated dUTP nick end labeling (TUNEL) assays. Increased green nuclear fluorescence reflects DNA degradation and apoptosis of LNT-229 glioblastoma cells treated with doxazosin for 24 h, compared to solvent controls. (E, F) Microscopic findings after treatment of LNT-229 cells with vehicle (E) or 30 µM doxazosin (F) further illustrate doxazosin-associated cell death. Scale bars, 100 µm. (G) Concentration-response relationship obtained from 2,3-bis-(2-methoxy-4-nitro-5-sulfophenyl)-2H-tetrazolium-5-carboxanilide salt (XTT) cell viability assays reveals an EC_50_ value of 35.3 µM in LNT-229 cells. (H) Time course of doxazosin-associated apoptosis. Cell death was determined using the XTT-based assay (n = 3 to 6 assays). Data are represented as mean ± SEM.

Apoptosis of LNT-229 cells was analyzed *in situ* by TUNEL (terminal deoxyribonucleotide transferase-mediated dUTP nick end labeling) fluorescence, assessing DNA damage and fragmentation as characteristic apoptotic features. Compared to baseline conditions (Figures 1A and 1E), increased apoptosis rates were detected following administration of the hERG inhibitor and α_1_-adrenoceptor antagonist, doxazosin (10–50 µM), for 24 h (Figures 1B to 1D, 1F). Application of 10 µM doxazosin achieved significant hERG current reduction *in vitro* in human embryonic kidney (HEK) cells that were either stably (>95% inhibition) or transiently transfected with hERG cDNA (∼90% inhibition) [14], [15]. Quantification of GB cell death using a XTT-based cell viability assay revealed a half-maximal effective doxazosin concentration of 35.3±5.2 µM (n = 4–6 independent assays; Figure 1G). 50 µM doxazosin reduced cell viability to 14.1±10.8% (n = 6; p = 0.003). The treatment duration required to achieve half-maximal pro-apoptotic effects of 10, 20, and 30 µM doxazosin was 48.8±1.2 h, 26.2±2.8 h, and 29.6±1.0 h, respectively (n = 3–6 assays; Figure 1H).

**Figure 2:**
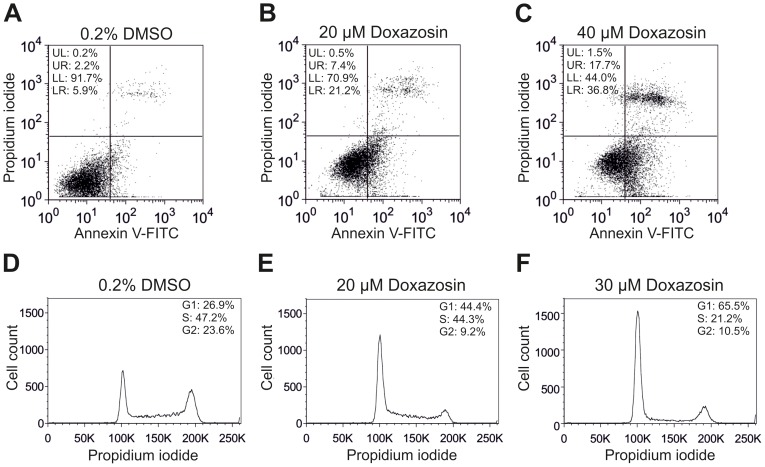
Quantification of apoptosis and cell cycle arrest in LNT-229 glioblastoma cells. (A–C) Representative flow cytometric analyses indicate pro-apoptotic effects of doxazosin. Cells were treated with doxazosin for 24 h and analyzed by fluorescent annexin V labeling to detect phosphatidylserine (PS) externalization as early and specific apoptotic feature. Early apoptotic cells are located in the lower right (LR) quadrant. Propidium iodide co-staining served to indicate late apoptotic cells (upper right quadrant). (D–F) Cell cycle arrest associated with doxazosin treatment (24 h), demonstrated by flow cytometry. Propidium iodide fluorescence intensity correlates with the amount of cellular DNA content. Decreased diploid DNA content reflects a reduced number of cells in G2 and M phases after doxazosin application (E, F) compared to solvent controls (D), whereas the fraction of haploid cells in the G0/G1 phase was elevated. Results and data from representative experiments are shown.

Specific quantification of drug-induced apoptosis was performed using labeled annexin V to detect phosphatidylserine (PS) translocation to the outer leaflet of the plasma membrane as early apoptotic feature. The fraction of early apoptotic GB cells determined after 24 h increased from 5.7±0.6% (solvent control; Figure 2A) to 20.3±7.6% (p = 0.031) and 33.6±4.0% (p = 0.011) following application of 20 µM and 40 µM doxazosin, respectively (Figures 2B and 2C; n = 3–4 assays). In addition, propidium iodide co-staining revealed that the fraction of late apoptotic cells yielded 11.9±4.7% (20 µM; n = 4; p = 0.033; Figure 2B) and 20.6±3.9% (40 µM; n = 3; p = 0.025; Figure 2C) after treatment with doxazosin compared to 3.1±1.2% (n = 4) under control conditions (Figure 2A).

### Doxazosin Causes Cell Cycle Arrest in the G0/G1 Phase

In addition to apoptosis, we examined the effects of doxazosin on cell cycle regulation of LNT-229 cells. Cell cycle phases were distinguished by flow cytometry after doxazosin treatment for 24 h (Figures 2D to 2F). The fraction of cells in the G0/G1 phase was enhanced by doxazosin from 28.2±2.6% (solvent control; Figure 2D) to 43.7±1.1% (20 µM doxazosin; p = 0.008; Figure 2E) and 64.8±1.8% (30 µM doxazosin; p = 0.023; Figure 2F) (n = 3 assays). In contrast, GB cells in the G2/M phase were reduced from 23.1±1.6% to 8.1±1.4% (p = 0.016) and 11.4±1.8% (p = 0.012) under these conditions (n = 3), reflecting cell cycle arrest and anti-proliferative effects of the drug.

### Doxazosin Triggers Apoptosis of U87MG Glioblastoma Cells

**Figure 3:**
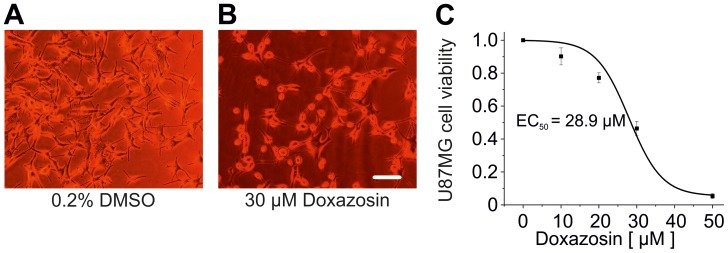
Apoptosis of U87MG human glioblastoma cells induced by doxazosin. (A, B) Representative microphotographs illustrate reduced viability of U87MG cells treated with 30 µM doxazosin (24 h) compared to controls exposed to the solvent, 0.2% DMSO. The scale bar represents 20 µm. (C) Concentration-response relationships based on data obtained using XTT assays for U87MG cell death yield an EC_50_ value of 28.9 µM. Data are given as mean ± SEM.

Pro-apoptotic effects of doxazosin were investigated in U87MG glioblastoma cells to exclude LNT-229-specific cellular effects. GB cell death (Figures 3A and 3B) was quantified using the XTT assay following doxazosin application (24 h), yielding a half-maximal effective concentration of 28.9±1.3 µM (n = 4–6 independent assays; Figure 3C).

### Desipramine Induces Death of LNT-229 Cells Similar to Doxazosin

**Figure 4:**
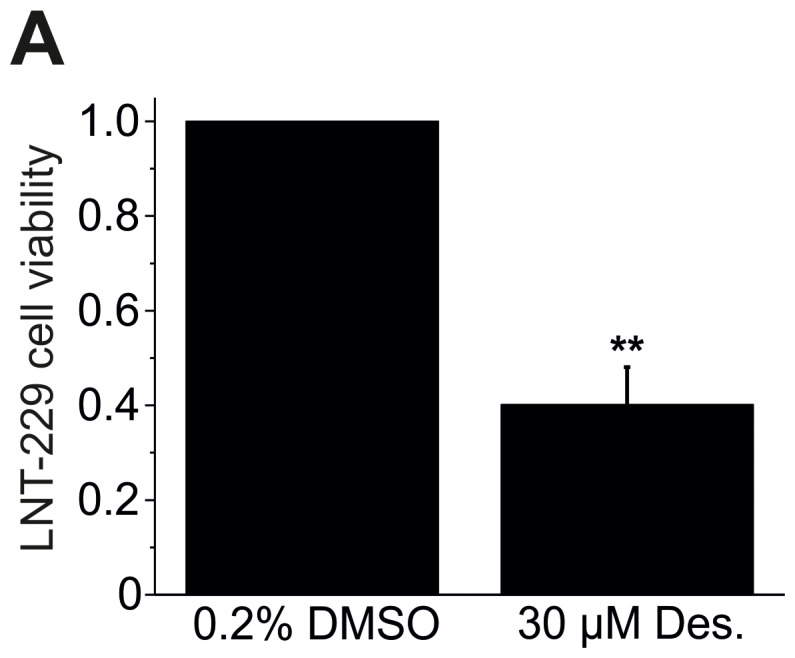
Desipramine induces LNT-229 cell apoptosis. Desipramine (Des.) treatment (30 µM; 24 h) significantly reduced LNT-229 cell viability quantified using the XTT assay. Mean (± SEM) data are shown (n = 3 assays; **p<0.01).

To further assess pro-apoptotic effects of hERG antagonism, we applied the antidepressant drug desipramine that has previously been shown to reduce hERG currents and prevent hERG protein trafficking to the plasma membrane [11]. LNT-229 cells were treated with 30 µM desipramine for 24 h (Figure 4). This concentration achieved ∼70% reduction of hERG protein and ∼98% decrease of hERG currents in human embryonic kidney cells [11]. Desipramine reduced cell viability quantified using the XTT assay to 42.5±8.1% compared to control cells (n = 3; p = 0.008).

### Pro-apoptotic Signaling in LNT-229 Cells

**Figure 5:**
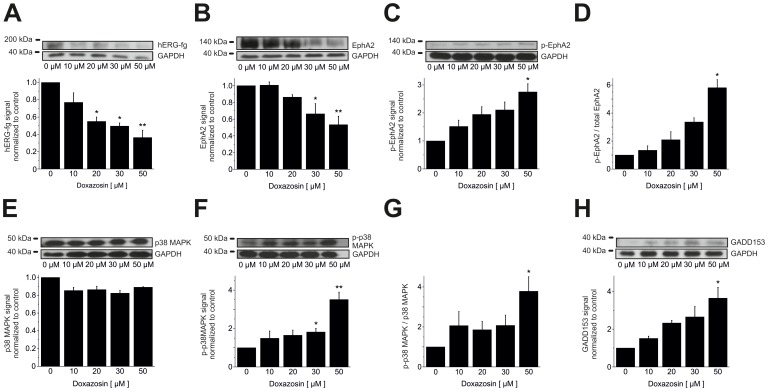
Apoptotic signaling in LNT-229 cells. (A) Human ether-a-go-go-related gene (hERG) K^+^ channels exhibited reduced expression in the presence of doxazosin. (B) Doxazosin-induced apoptosis was further associated with lower erythropoietin-producing human hepatocellular carcinoma receptor tyrosine kinase A2 (EphA2) protein levels. Enhanced phosphorylation (i.e. activation) of EphA2 (C, normalized p-EphA2 levels; D, p-EphA2 relative to total EphA2) and of p38 mitogen-activated protein kinase (MAPK; F, normalized p-p38 MAPK levels; G, p-p38 MAPK relative to total p38 MAPK) was observed, while total p38 MAPK levels were not significantly affected by doxazosin (E). Finally, growth arrest and DNA damage inducible gene 153 (GADD153) protein levels were elevated upon doxazosin treatment (H). Original Western Blots and mean data normalized to control conditions obtained from n = 3 independent assays (± SEM) are provided (*p<0.05; **p<0.01). GAPDH, glyceraldehyde-3-phosphate dehydrogenase.

**Figure 6:**
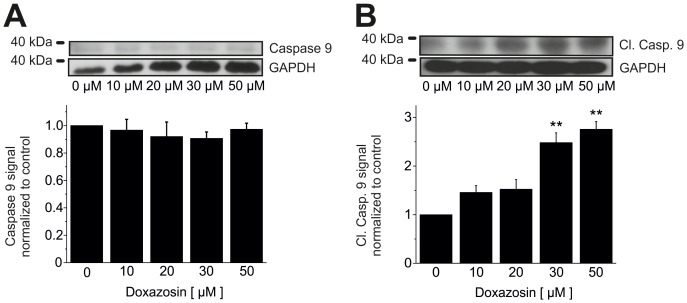
Caspase 9 activation *en route* to apoptosis. (A, B) Increased levels of cleaved caspase 9 and unaffected total caspase 9 protein expression were detected in LNT-229 cells treated with doxazosin. Representative Western blots and mean (± SEM) optical densities normalized to doxazosin-free conditions are presented for cells exposed to increasing concentrations of doxazosin (n = 3 independent assays; **p<0.01; GAPDH, glyceraldehyde-3-phosphate dehydrogenase).

Pro-apoptotic signaling pathways were elucidated in detail in human LNT-229 glioblastoma cells following doxazosin treatment (24 h application time). Apoptosis induced by doxazosin was associated with reduction of fully glycosylated, mature hERG ion channel protein (Figure 5A) that has previously been implicated in apoptosis regulation [11]–[16]. HERG protein levels decreased significantly in apoptotic LNT-229 cells following doxazosin treatment (50 µM) by 63.7±8.4% (n = 3; p = 0.003). In addition, the level of Tyr-593-phosphorylated (activated) pro-apoptotic erythropoietin-producing human hepatocellular carcinoma receptor tyrosine kinase A2 (EphA2) increased after doxazosin treatment (50 µM) by 2.8±0.29-fold (n = 3; p = 0.048; Figure 5C). The EphA2 phosphorylation state relative to total EphA2 protein was enhanced as well by 5.8±0.57-fold (n = 3; p = 0.046; Figure 5D), while total EphA2 protein was reduced by 53.3±10.1% (n = 3; p = 0.003; Figure 5B). Phosphorylation and down-regulation of EphA2 cause cell death in tumors and cardiac cells [19], [25], [26]. LNT-229 cell apoptosis was further associated with increased phosphorylation and activation of pro-apoptotic p38 mitogen-activated protein kinase (MAPK) by 3.5±0.38-fold (n = 3; p = 0.005; Figure 5F). The ratio of activated p-p38 MAPK *versus* total p38 MAPK protein was increased by 3.8±0.73-fold (n = 3; p = 0.024; Figure 5G), whereas total p38 MAPK levels were not changed by 50 µM doxazosin (−11.0±0.6%; n = 3; p = 0.20; Figure 5E). P38 MAPK activation resulted in 3.6±0.57-fold increased protein expression of the pro-apoptotic nuclear transcription factor growth arrest and DNA damage inducible gene 153 (GADD153) (n = 3; p = 0.015) in the presence of 50 µM doxazosin (Figure 5H). Pro-apoptotic signaling in GB cells involved activation of the initiator caspase 9 by 2.8±0.16-fold through cleavage (n = 3; p = 0.005), whereas expression of full-length caspase 9 was not modified (−2.7±4.4%; n = 3; p = 0.28) relative to control cells (Figures 6A and 6B).

**Figure 7:**
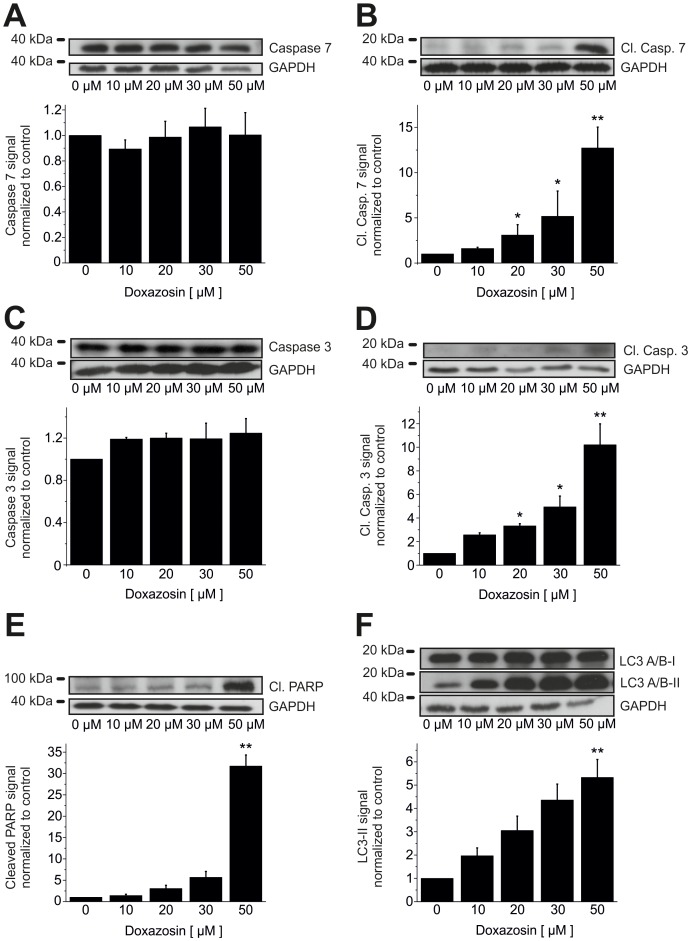
Downstream signaling in doxazosin-induced apoptosis. Doxazosin treatment induced cleavage of effector caspases 7 (B) and 3 (D), associated with unaltered expression of pre-processed caspase 3 and 7 levels in LNT-229 cells (A, C). (E) Enhanced cleavage (inactivation) of poly-ADP-ribose-polymerase (PARP) was observed with increasing concentrations of doxazosin, representing a characteristic apoptotic step. (F) Autophagy-associated phagocytosis of dead cells is indicated by increased levels of the lower migrating form of microtubule-associated protein 1 light chain 3 (LC3). Typical Western blots and mean (± SEM) optical densities normalized to drug-free conditions are shown (n = 3 independent assays; *p<0.05; **p<0.01; GAPDH, glyceraldehyde-3-phosphate dehydrogenase).

Effector caspases 7 (by 12.7±2.3-fold; n = 3; p = 0.002; Figure 7B) and 3 (by 10.2±1.8-fold; n = 3; p = 0.002; Figure 7D) were similarly activated. Expression of corresponding full-length caspase 7 (+0.3±17.5%; n = 3; p = 0.83) and caspase 3 protein (+24.4±13.7%; n = 3; p = 0.18) was not significantly affected by 50 µM doxazosin (Figures 7A and 7C). Poly-ADP-ribose-polymerase (PARP) represents an established target of caspases 7 and 3 *en route* to apoptosis. We observed cleavage and 31.7±2.6-fold inactivation of PARP by 50 µM doxazosin relative to control cells (n = 3; p = 0.009; Figure 7E). Finally, 5.3±0.77-fold increased generation of the lower migrating form of microtubule-associated protein 1 light chain 3 (LC3) indicates autophagy-associated phagocytosis and clearance of dead cells (n = 3; p = 0.003; Figure 7F).

### SiRNA Inactivation of hERG K^+^ Channels Triggers Apoptosis of GB Cells

**Figure 8:**
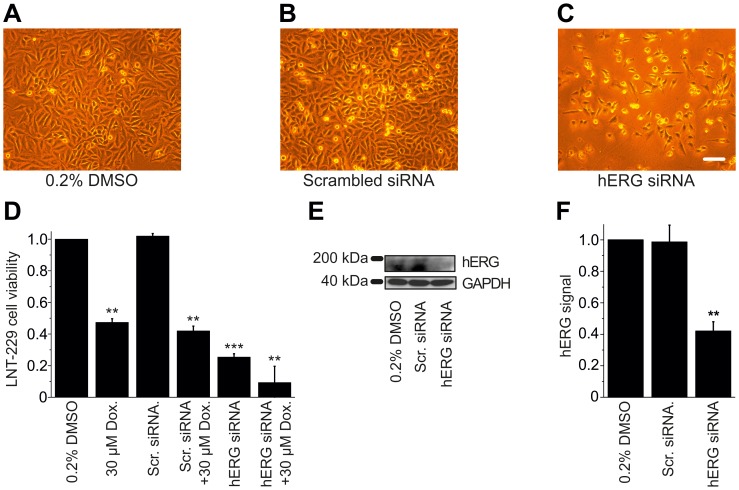
Knock down of hERG channel protein mimics pro-apoptotic effects of doxazosin. (A–C) Representative microphotographs reflect viability of LNT-229 cells treated with small interfering RNA (siRNA) to suppress hERG expression (C) compared to controls exposed to DMSO (solvent; A) or scrambled siRNA (B). Scale bar, 100 µm. (D) XTT assay-based quantification of apoptotic cells following indicated treatment normalized to solvent controls (n = 4 to 6 assays;). Reduced viability associated with doxazosin or hERG siRNA treatment was further increased by simultaneous application of both compounds. (E, F) Western blot analysis (E) and corresponding mean hERG expression levels normalized to GAPDH expression (F) confirmed hERG protein knock down by ∼58%. Data are given as mean ± SEM (**p<0.01; ***p<0.001). Scr, srambled siRNA; GAPDH, glyceraldehyde-3-phosphate dehydrogenase.

We next sought to explore the mechanistic significance of reduced hERG protein levels in LNT-229 cell apoptosis. To this end, hERG siRNA was applied to specifically knock down hERG expression prior to XTT-based viability assessment. Compared to control cells treated with the solvent (0.2% DMSO; n = 4; Figures 8A and 8D), hERG siRNA reduced cell viability to 25.4±2.1% (n = 5; p = 0.0008; Figures 8C and 8D), whereas scrambled siRNA (24 h) did not significantly affect cell death (n = 4; p = 0.26; Figures 8B and 8D). When applied simultaneously, doxazosin (30 µM) and hERG siRNA exhibited additive effects on GB cell apoptosis, resulting in reduction of cell viability to 9.2±10.3% (n = 3; p = 0.005; Figure 8D). Successful siRNA-mediated suppression of hERG protein expression to 41.2±7.3% (n = 3; p = 0.003) compared to cells exposed to the solvent was demonstrated by immunoblot analysis (Figures 8E and 8F). Control cells treated with scrambled siRNA did not exhibit any reduction of hERG protein (98.7±10.7% of control expression; n = 3; p = 0.69). Apoptosis rates associated with doxazosin treatment (30 µM) (47.4±2.1%; n = 4; p = 0.002; Figure 8D) were not modified by scrambled siRNA (41.9±3.0%; n = 4; p = 0.003; Figure 8D).

### Modulation of Doxazosin-induced Apoptosis by Terazosin, a Small Molecule hERG Inhibitor

**Figure 9:**
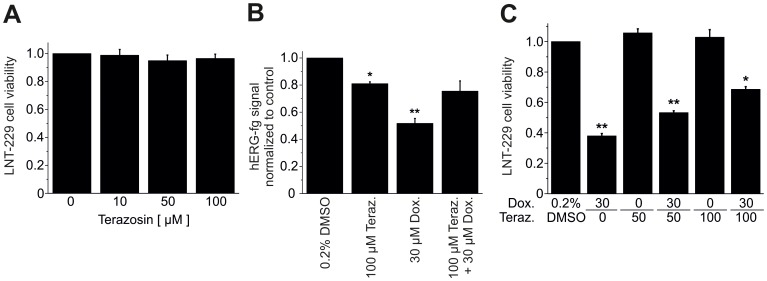
The small molecule hERG inhibitor terazosin protects GB cells from doxazosin-induced apoptosis. (A, B) Terazosin treatment did not impair LNT-229 cell viability (A) and caused less suppression of hERG protein compared to doxazosin (B). Combined drug exposure attenuated doxazosin-associated hERG protein suppression (B). Cell death quantified using the XTT assay was inhibited in concentration-dependent fashion (C). Mean (± SEM) data are presented (n = 4 to 6 assays; *p<0.05; **p<0.01).

The mechanistic role of small molecule-induced hERG protein suppression in apoptosis of GB cells was tested using terazosin, an α_1_-adrenoceptor antagonist that reduces hERG currents in culture cells [14]. Terazosin was applied to LNT-229 cells for 24 h. In contrast to doxazosin (Figure 1), terazosin at concentrations between 10 µM to 100 µM did not significantly affect cell viability quantified by XTT assay (n = 5 to 6 cells; Figure 9A) despite ∼90% blockade of hERG current at 100 µM terazosin [14]. Relative cell viability yielded 96.6±3.1% (n = 6; p = 0.86) after application of 100 µM terazosin. This observation supports a role for hERG protein expression in GB cell apoptosis independent of hERG ion channel function. Consistent with this notion, terazosin (100 µM; 24 h) exhibited weak effects on hERG protein levels in LNT-229 cells (−20.4±2.8%; n = 3; p = 0.03), while doxazosin (30 µM; 24 h) suppressed hERG expression by 48.2±1.7% (n = 3; p = 0.002; Figure 9B). However, application of 30 µM doxazosin +100 µM terazosin did not result in additive reduction of hERG protein. An unexpected finding was revealed: simultaneous application of 100 µM terazosin attenuated doxazosin-induced hERG suppression to 24.5±11.4% (n = 3; p = 0.07; Figure 9B). This observation suggests that hERG receptor occupancy interferes with binding of terazosin. Antagonism of doxazosin binding by terazosin was associated with restoration of protein expression and increased survival of GB cells. LNT-229 cell viability was increased by co-application of 50 µM and 100 µM terazosin to 53.3±4.8% (n = 3; p = 0.008) and 66.7±6.9% (n = 4; p = 0.02), compared to application of 30 µM doxazosin for 24 h (41.0±1.5%; n = 4; p = 0.002; Figure 9C).

## Discussion

### Pro-apoptotic and Anti-proliferative Functions of Doxazosin in Human Glioblastoma Cells

We identified and characterized doxazosin, an α_1_-adrenoceptor antagonist clinically used to treat hypertension and benign prostatic hyperplasia, as a novel small molecule trigger of apoptosis in GB cells. Doxazosin induced apoptosis of human GB cells in time- and concentration-dependent fashion. Apoptotic cell death was confirmed using TUNEL- and annexin V-based assays to demonstrate DNA fragmentation and phosphatidylserine translocation to the outer leaflet of the plasma membrane as characteristic apoptotic features. In addition, concentration-dependent activation of specific pro-apoptotic signaling (i.e., cleavage of PARP and of caspases 3, 7, and 9) was observed in the presence of doxazosin. Suppression of mature hERG protein by doxazosin was identified as crucial molecular event in GB cell apoptosis. Pro-apoptotic effects were similarly observed with desipramine, an antidepressant drug that induces reduction of hERG protein [11].

Doxazosin treatment resulted in activation of the pro-apoptotic receptor tyrosine kinase EphA2 and associated degradation of EphA2 that is characteristic to activated receptor tyrosine kinases, consistent with data obtained from prostate cancer (PC3) and breast cancer (MDA-MB-231) cells [27]. Doxazosin stimulation also activated pro-apoptotic factors p38 MAPK and GADD153. Furthermore, initiator caspase 9 and effector caspases 7 and 3 were activated in response to doxazosin treatment. PARP inactivation reflected loss of DNA repair activity. Finally, phagocytosis of dead cells was indicated by increased expression of the lower migrating form of LC3 protein. The correlation of doxazosin application with established pro-apoptotic signaling reported in this work requires mechanistic validation in future studies.

Pro-apoptotic effects were accompanied by GB cell cycle arrest in the G0/G1 phase that shows anti-proliferative actions of doxazosin. Similar effects were observed in GH3 and AtT-20 pituitary tumor cells and attributed to attenuated nuclear factor-κB signaling [28]. Moreover, hERG protein reduction through siRNA knock down decreased proliferation in small cell lung cancer cells independent of the ion-conducting function of the channel [29].

### The Mechanistic Role of hERG K^+^ Channel Protein

A pivotal role of hERG protein expression for apoptosis of GB cells is suggested by reduced hERG expression following doxazosin treatment. Similar reduction of cell viability observed with desipramine further supports mechanistic significance of hERG protein levels in GB cell apoptosis. Furthermore, specific siRNA-mediated knock down of hERG expression triggered apoptosis, confirming the mechanistic role of small molecule-induced hERG protein suppression. Finally, antagonism of doxazosin-induced hERG protein suppression and glioblastoma cell death by co-treatment of terazosin and doxazosin indicates the functional relevance of hERG protein in GB cell apoptosis. In contrast to doxazosin, terazosin did not induce apoptosis despite similar degrees of acute hERG K^+^ channel blockade *in vitro* (doxazosin, >95%; terazosin, ∼90%; [14]). Mechanistic differentiation of acute blockade and apoptosis indicates a role for hERG protein expression independent of hERG function. Doxazosin and other hERG K^+^ channel ligands impair protein expression by selective disruption of hERG trafficking into the cell surface membrane [10], [11], [15]. This mechanism of action occurs mechanistically independent of acute hERG current blockade, a well-established class III antiarrhythmic mode of action and pro-arrhythmic property of several non-antiarrhythmic compounds [30]. The presence of independent drug-channel interaction sites for inhibition of hERG forward trafficking and acute blockade is supported by the observation that certain compounds induce current block with little or no trafficking inhibition (and *vice versa*) [10], [20]. Moreover, elimination of a common drug binding site formed by aromatic residues in the hERG channel pore [31] abolished acute current block without affecting protein trafficking inhibition (reviewed in [10]). Here, protection from GB cell apoptosis by terazosin that exhibits low intrinsic trafficking inhibition may be readily explained by pharmacological “rescue” of hERG surface protein expression through competitive binding of doxazosin and terazosin at a common trafficking receptor site. Treatment with terazosin prevented doxazosin binding and preserved hERG protein expression. However, an allosteric interaction between separate doxazosin and terazosin binding sites cannot be excluded, and the precise molecular mechanism underlying the suppression of mature hERG protein in doxazosin-associated apoptosis remains to be elucidated.

In addition to its interaction with hERG channels, doxazosin may directly bind and activate EphA2 [27]. We detected high apoptosis rates following specific siRNA knock down of hERG protein that were increased further by ∼16% through additional application of doxazosin. This difference may be partially mediated through direct activation of EphA2 signaling by doxazosin. Furthermore, additional reduction of hERG viability in the presence of anti-HERG siRNA may be caused by doxazosin-induced suppression of residual hERG protein (∼42%).

In summary, there are multiple lines of evidence that indicate and confirm the mechanistic significance of hERG channels in GB cell apoptosis:

1. Previous data revealed that doxazosin specifically induced apoptosis in hERG-expressing cells [15].
2. Concentration-dependent hERG protein reduction was observed in apoptotic cells exposed to doxazosin (Figure 5A).
3. The antidepressant drug desipramine that blocks hERG protein trafficking to the plasma membrane [11] exhibited pro-apoptotic effects similar to doxazosin (Figure 4).
4. Anti-hERG siRNA reduced hERG protein expression and induced apoptosis similar to doxazosin (Figure 8).
5. Terazosin did not reduce hERG protein expression (Figure 9B) and did not show pro-apoptotic effects (Figure 9A) despite structural similarities between terazosin and doxazosin.
6. In the presence of doxazosin, terazosin rescued hERG protein expression and protected from apoptosis (Figure 9C).

### Clinical Implications and Future Directions

Current treatment of GB is associated with low patient survival rates owing to uncontrolled proliferation and resistance to apoptosis or cytotoxic treatment [2], [3]. This work identifies doxazosin as small molecule compound that triggers apoptosis and exerts anti-proliferative effects in human GB cells. Thus, we propose that the Food and Drug Administration (FDA)- and European Medicines Agency (EMA)- approved drug doxazosin may be re-purposed for treatment of human glioblastoma in the future following *in vivo* concept validation. In patients treated with doxazosin for hypertension or benign prostatic hyperplasia, plasma levels between 42 nM and 244 nM have been reported [32]–[34] with 98.3% protein binding [35]. These data indicate that apoptosis of GB cells will not be induced using doses that are currently applied for non-malignant diseases. Rather, increased doxazosin doses would be required for the use as anticancer drug that have not yet been determined *in vivo*.

More widespread expression of hERG protein in tumors (e.g. colorectal or endometrial cancer) and cancerous cells (e.g. acute myologenous or chronic lymphocytic leukemia) [12] suggests potential significance of hERG-based anticancer therapy beyond glioblastoma that warrants further exploration, carefully considering the significance of the individual cellular context for regulation of cell death and proliferation. A systematic *in vivo* investigation of anticancer properties and potential cardiac side effects of small molecule-induced hERG protein suppression is required. Specifically, significant apoptosis of cardiac myocytes and marked inhibition of repolarizing hERG channels have been observed at similar concentrations compared to induction of GB cell apoptosis [13]–[15]. Thus, proarrhythmic and cardiotoxic risks of hERG inhibitors and associated cardiac *I*_Kr_ current reduction/QTc interval prolongation or apoptosis of cardiac myocytes require careful pre-clinical evaluation and clinical monitoring when applying doxazosin and derivatives in clinical oncology [6], [8], [10], [11], [16], [19], [20], [36].

## Conclusions

The small molecule compound doxazosin induces apoptosis in GB cells and exhibits anti-proliferative function. HERG potassium channels, previously recognized to regulate cardiac action potential repolarization, modulate GB cell apoptosis. The data imply that doxazosin-related reduction of hERG protein expression may be linked to increased apoptosis rates. This novel finding of apoptosis regulation in GB cells may provide new options for anticancer therapies. Particularly, doxazosin and doxazosin-based derivatives may be useful as novel treatment for GB due to their pro-apoptotic and anti-proliferative properties.
